# Test for Arsenic

**Published:** 1842-08-13

**Authors:** 


*Test for Arsenic.*—Mr. Rodgers, at a recent meeting of the Royal Medico-Botanical Society, mentioned the following test for arsenic:—The galvanic test was first proposed by the German chemists, who employed a simple apparatus, similar to that used for electrotype purposes, placing the suspected solution in the inner vessel, and a solution of hydrochlorate of ammonia in the outer; they then put a piece of zinc in the outer vessel, and bent a platinum wire (attached to the zinc) so as to dip into the inner one. In the course of a few hours the arsenic was deposited on the wire.—*Provin. Med. Jour.* June 4, 1842.

